# Effect of metallo-β-lactamase production and multidrug resistance on clinical outcomes in patients with *Pseudomonas aeruginosa* bloodstream infection: a retrospective cohort study

**DOI:** 10.1186/1471-2334-13-515

**Published:** 2013-11-01

**Authors:** Matthias Willmann, Ines Kuebart, Matthias Marschal, Klaus Schröppel, Wichard Vogel, Ingo Flesch, Uwe Markert, Ingo B Autenrieth, Florian Hölzl, Silke Peter

**Affiliations:** 1 Institute of Medical Microbiology and Hygiene, University of Tübingen, Elfriede-Aulhorn-Str. 6, 72076 Tübingen, Germany; 2 Medical Center, Department of Hematology, Oncology, Immunology, Rheumatology & Pulmonology, University of Tübingen, Tübingen, Germany; 3 BG Trauma Center, University of Tübingen, Tübingen, Germany; 4 Clinic for General, Visceral and Vascular Surgery, Zollernalb Hospital, Albstadt, Germany

**Keywords:** Bacteremia, Antimicrobial therapy, Mortality, Prognosis, Length of stay, MBL

## Abstract

**Background:**

Blood stream infections (BSI) with *Pseudomonas aeruginosa* lead to poor clinical outcomes. The worldwide emergence and spread of metallo-β-lactamase (MBL) producing, often multidrug-resistant organisms may further aggravate this problem. Our study aimed to investigate the effect of MBL-producing *P. aeruginosa* (MBL-PA) and various other resistance phenotypes on clinical outcomes.

**Methods:**

A retrospective cohort study was conducted in three German hospitals. Medical files from 2006 until 2012 were studied, and a number of 113 patients with *P. aeruginosa* BSI were included. The presence of VIM, IMP and NDM genes was detected using molecular techniques. Genetic relatedness was assessed through multilocus sequence typing (MLST). The effect of resistance patterns or MBL production on clinical outcomes was investigated by using multivariate Cox regression models.

**Results:**

In-hospital mortality was significantly higher in patients with MBL-PA and multidrug-resistant *P. aeruginosa*. However, neither BSI with MBL-PA nor BSI with various resistance phenotypes of *P. aeruginosa* were independently associated with mortality or length of hospital stay. In multivariate models, the SAPS II score (HR 1.046), appropriate definitive treatment (HR range 0.25-0.26), and cardiovascular disease (HR range 0.44-0.46) were independent predictors of mortality. Concomitant infections were associated with an excess length of stay (HR < 1).

**Conclusions:**

Medication with appropriate antimicrobial agents at any time during the course of infection remains the key for improving clinical outcomes in patients with *P. aeruginosa* BSI and should be combined with a strict implementation of routine infection control measures.

## Background

*Pseudomonas aeruginosa* is an important nosocomial pathogen [1]. Blood stream infections, primarily observed in immunosuppressed individuals, are associated with high mortality [2,3]. The worldwide emergence of multidrug-resistant *P. aeruginosa* over the course of the last decades called the reliance on various antimicrobial agents into question [4-8]. The production of metallo-β-lactamase (MBLs) which confer resistance to all β-lactams except aztreonam is a mechanism of increasing clinical importance, largely driven by the international spread of MBL-producing organisms [9]. Therapeutic options against such strains are often severely compromised since other determinants of resistance tend to be linked to MBL genes on the same plasmid or integron structure [10,11]. Moreover, MBLs cannot be inhibited by currently approved β-lactamase inhibitors [12]. Several types of MBLs have been described, most importantly the IMP-type, VIM-type, and NDM-type enzymes [9].

Despite the growing body of epidemiological reports of metallo-β-lactamase-producing *P. aeruginosa* (MBL-PA) [8,9,13], more information about the clinical characteristics of MBL-PA infections is urgently needed. Most notably, the association between MBL-PA infection and clinical outcomes remains an unresolved issue. In two studies, a higher frequency of infection and fatal cases in patients with MBL-PA has been described [14,15]. Zavascki et al. also reported on a higher mortality among patients with MBL-PA nosocomial infections, but concluded that this result is most likely due to a delayed administration of appropriate treatment and the severity of the infections [16].

The aim of this study was to investigate the impact of MBL-production and different phenotypes of resistance on mortality and length of hospital stay in patients with *P. aeruginosa* blood stream infection and to shed some light on possible additional factors influencing such a relationship.

## Methods

### Setting and patients

The retrospective cohort study was performed from 1^st^ January 2006 until 31^st^ January 2012 in a 1500-bed tertiary teaching hospital, a 300-bed trauma center and a 500-bed district hospital in Tübingen, Germany, and the surrounding community. A broad spectrum of medical services is provided by these hospitals, including various surgical and medical specialties, pediatric units, dialysis and a maternity ward. Organ transplantations are performed at the tertiary teaching hospital. The study is reported pursuant to the STrengthening the Reporting of OBservational studies in Epidemiology (STROBE) guidelines [17]. The study has been approved by the local research ethics committee of the University of Tübingen (reference number: 035/2012R).

### Study design and definitions

Adult patients (≥ 18 years) suffering from a blood stream infection (BSI) with ≥ 1 blood culture positive for *P. aeruginosa* were considered eligible. Patients were excluded if they were not admitted to the hospital. Hybase software (Tieto GmbH, Eschborn, Germany) was used to retrospectively identify eligible patients from the laboratory information system. Every patient was included only once at the time of the first positive blood culture (index culture).

In-hospital mortality for any cause and length of hospital stay were the primary outcomes, while exposure to strains of *P. aeruginosa* producing an MBL enzyme or to isolates exhibiting different resistance phenotypes was the variable of interest. Multidrug-resistant *P. aeruginosa* (3/4MDR-PA) was defined as resistant to at least three of the following antimicrobial agents: piperacillin-tazobactam; ceftazidime; meropenem; and ciprofloxacin [18]. For the definition of multidrug resistance we considered antimicrobial agents which have a bactericidal effect on *P. aeruginosa* and can be administered as effective monotherapy. Thus, aminoglycosides were not a part of the definition [18]. Intermediately susceptible isolates were deemed resistant. MBL-PA was defined as MBL-producing organism regardless of the susceptibility pattern.

Patient files were reviewed by medically trained personnel. Clinical data obtained included age; sex; site of infection (primary, secondary, vascular catheter-related) according to the definition of the International Sepsis Forum [19]; nosocomial infection (defined as infection that occurred ≥ 48 hours after hospital admission); baseline diseases; Charlson comorbidity score at admission [20]; immunosuppression, such as HIV and/or neutropenia (neutrophil count ≤ 1000 cell/μl) and/or immunosuppressive chemotherapy within the previous two months (anti-cancer drugs and anti-inflammatory monoclonal antibodies) and/or receipt of steroids (prednisolone ≥ 10 mg/daily or equivalent dose); previous surgery during hospitalization; and the presence of concomitant infections with relevant pathogenic organisms other than *P. aeruginosa*. The individual physiological condition was assessed by determining the simplified acute physiology score II (SAPS II) of the index culture day [21]. If a SAPS II parameter was not available for the index culture day, it was gained from the time point closest to the index culture day (± 48 hours).

Appropriate antimicrobial treatment was defined as systemic administration of at least one antimicrobial agent to which the isolate was *in vitro* susceptible. Monotherapy with aminoglycosides was not considered appropriate despite *in vitro* susceptibility. Appropriate antimicrobial treatment was categorized in i) appropriate empirical treatment (AET) which was administered within 24 hours after samples for blood cultures were drawn, and ii) appropriate definitive treatment (ADT) which was administered at any time after the index culture.

### Species identification and phenotypic testing

Species identification was performed through a linear MALDI-TOF mass spectrometer (AXIMA Assurance, bioMérieux, Marcy l’Etoile, France, Saramis Database Version 4.09), supplemented by Vitek 2 system identification (bioMérieux, Marcy l’Etoile, France). *In vitro* bacterial susceptibility testing of *P. aeruginosa* isolates was performed by use of disk diffusion tests following the EUCAST guidelines [22-24]. Colistin susceptibility was interpreted according to the CLSI breakpoints [25]. For MBL detection, a combined disk test with EDTA was performed on all isolates as previously described [26]. Briefly, antibiotic disks (Becton Dickinson GmbH, Heidelberg, Germany) containing 10 μg meropenem alone and in combination with 930 μg EDTA were used. An increase of ≥ 7 mm in zone diameter in the presence of EDTA compared to the meropenem disk alone was considered an indication of possible MBL production.

### PCR assays and DNA sequencing

*P. aeruginosa* isolates having a reduced meropenem zone size (defined as < 24 mm) and/or being positive in the EDTA combined disk test were further investigated for the presence of MBL genes [26]. Whole cell DNA was used as a template in PCR assays. For the simultaneous detection of *bla*_VIM_ and *bla*_IMP_ genes, a multiplex PCR amplification was performed according to a protocol described elsewhere [26]. The entire VIM and IMP genes were sequenced using the primer pairs IMP-A–IMP-B or VIM2004A-VIM2004B in combination with the class 1 integron primer pair 5CS and 3CS or alternatively VIM-2SQR [26,27]. Isolates negative for VIM and IMP genes that exhibited reduced meropenem and ceftazidime zone sizes (defined as < 24 mm and < 16 mm, respectively) were further investigated for the presence of NDM and class A carbapenemase (KPC and GES) genes at the National Reference Laboratory for Multidrug-resistant Gram-negative Bacteria (Bochum, Germany) by using molecular detection techniques.

### Multilocus sequence typing (MLST)

MLST was performed on the 3/4MDR-PA including all MBL-PA in accord with the instructions on the *P. aeruginosa* MLST web site (http://pubmlst.org/paeruginosa/). Internal fragments of seven housekeeping genes (*acsA*, *aroE*, *guaA*, *mutL*, *nuoD*, *ppsA* and *trpE*) were amplified and subsequently sequenced to determine the sequence type.

### Statistical analysis

D’Agostino’s K-squared test was used to check continuous variables for normality. The Student t-test was used for comparison when data were normally distributed. The Wilcoxon rank sum test was performed when transformation of variables could not achieve a normal distribution of values. A chi-squared test or–when appropriate–the Fisher’s exact test were employed to compare differences in proportions.

Cox regression was used to model the relationship between the exposure (blood stream infection with MBL-PA or a certain resistance type of *P. aeruginosa*) and the two outcomes (in-hospital mortality and length of stay) by calculating the hazard ratio for death and the hazard ratio for discharge (dead or alive). Exposed patients were compared with the rest of the cohort. To determine the effect on morbidity, the length of stay (LOS) was considered as failure variable. A hazard ratio of discharge <1 indicates a smaller hazard of being discharged and therefore an excess of stay adjusted for the length of stay before the index day (time-adjusted model) and additional confounders (fully-adjusted model) [28]. Hypothesis testing was performed by using the likelihood ratio test. Model identification for multivariate analysis was accomplished as described elsewhere [29]. Briefly, any variable with a P-value of < 0.2 in univariate analysis was incorporated in multivariate models, wherein only variables with a P-value of ≤ 0.1 were retained. Excluded variables were subsequently tested for confounding by adding them one at a time to the identified model. In case of substantial confounding (defined as a change in the models’ coefficients greater than 10%) they were included into the final model. The exposure of interest was always retained in the model, regardless of the p-value. Potential interactions were examined through the likelihood ratio test. The proportional hazards assumption was verified on the basis of Schoenfeld residuals.

All analyses were carried out by using Stata version 12.0 (Stat Corp., College Station, TX, USA). A P-value < 0.05 (two-sided) was deemed significant.

## Results

### MBL detection and susceptibility profiles

Over the course of the six year study a total of 6064 patients with bacterial BSI were identified. Of these, 120 patients (2%) had a PA-BSI. Three patients were excluded because they were not admitted to the hospital. Of the remaining 117 study entrants, four patients were excluded from the analysis because clinically relevant data were missing. The remaining 113 patients were analyzed.

A number of 34 isolates met the criteria for a possible MBL production and were subsequently tested for the presence of MBL genes. Eighteen isolates (15.9%) that possessed a gene for a metallo-β-lactamase were detected. Four isolates were positive for VIM-2 (3.5%) and 14 isolates for IMP-8 (12.4%). The results of the susceptibility testing are shown in Table 1. All MBL-PA were susceptible to colistin but resistant to all other tested antimicrobials. Non-MBL-PA remained susceptible to a number of antimicrobial agents, particularly to ceftazidime (95%), amikacin (96%) and meropenem (85%). All *P. aeruginosa* isolates were susceptible to colistin.

**Table 1:** **Susceptibility profile of the 113** ***Pseudomonas aeruginosa*** **clinical isolates**

**Antimicrobial agent**	**MBL (n = 18), susceptible isolates (%)**	**Non-MBL (n = 95), susceptible isolates (%)**
Meropenem	0	85
Piperacillin-Tazobactam	0	83
Ciprofloxacin	0	78
Ceftazidime	0	95
Colistin	100	100
Amikacin	0	96
Fosfomycin	0	40

MBL, metallo-β-lactamase producer.

### Study population characteristics

Table 2 presents the basic characteristics of the study cohort. The median age was 64 years (IQR, 53-74 years) with 48% over 65 years. Male gender was predominant (63%). Frequent comorbidities were cardiovascular diseases (58%), diabetes (33%) and hematological cancer (32%). The most common sources of infection were the respiratory and urinary tract (19% and 16%, respectively). The source of infection was not identifiable in 44% of cases. The mean time to effective treatment was 1.05 days in our cohort.

**Table 2:** **Basic characteristics of 113 adult patients with** ***Pseudomonas aeruginosa*** **BSI**

**Parameter**	**Patients n (%)**
*Basic parameter*	
Age > 65 years	54 (48)
Male sex	71 (63)
Nosocomial infection	66 (58)
Fatal outcome	43 (38)
*Comorbid conditions*	
Diabetes	37 (33)
HIV	1 (1)
Hematological cancer	36 (32)
Cardiovascular disease	66 (58)
Pulmonary disease	11 (10)
Neurologic disease	24 (21)
Renal disease	11 (10)
Neutropenia	38 (34)
*Origin of bacteremia*	
Unknown (primary BSI)	50 (44)
Secondary BSI	55 (49)
Respiratory tract	21 (19)
Urinary tract	18 (16)
Intra-Abdominal	5 (4)
Surgical Site	1 (1)
Non-surgical site	10 (9)
VC BSI	8 (7)
*BSI pathogen*	
MBL-PA	18 (16)
3/4MDR-PA	27 (24)
MEM-resistant PA	32 (28)
CAZ-resistant PA	23 (20)
CIP-resistant PA	39 (35)
TZP-resistant PA	34 (30)

BSI, Bloodstream infection; HIV, Human immunodeficiency virus infection; VC BSI, vascular catheter-related blood stream infection; MBL-PA, metallo-β-lactamase producing *Pseudomonas aeruginosa*; 3/4MDR-PA, 3/4MDR-*Pseudomonas aeruginosa*; PA, *Pseudomonas aeruginosa*; MEM, Meropenem; CAZ, Ceftazidime; CIP, Ciprofloxacin; TZP, Piperacillin-tazobactam.

The in-hospital mortality was 38%. Differences in baseline characteristics between the two exposure groups MBL-PA and 3/4MDR-PA are shown in Table 3. Patients infected with a MBL-PA had a higher mortality than those with a Non-MBL-PA (61% vs. 34%, *P* = 0.03). A BSI with a 3/4MDR-PA resulted in a higher mortality as well (63% vs. 30%, *P* = 0.002). Interestingly, while patients with MBL-PA BSI overall had a higher SAPSII score (39.5 vs. 32.0, *P* = 0.002), there was merely a tendency towards a difference in the administration of definitive appropriate treatment in comparison to the Non-MBL-PA group. The opposite seemed to be true for patients with 3/4MDR-PA BSI: No significant difference in SAPSII was observed, but the number receiving appropriate definitive treatment was significantly lower (70% vs. 90%, *P* = 0.03). Patients infected with MBL-PA or 3/4MDR-PA were more frequently neutropenic (p < 0.001).

**Table 3:** **Baseline characteristics, comorbidities and treatment parameters of different multidrug-resistant** ***Pseudomonas aeruginosa***

**Parameter**	**MBL (n = 18)**	**Non-MBL (n = 95)**	**P-value**	**3/4MDR (n = 27)**	**Non-3/4MDR (n = 86)**	**P-value**
*Basic parameters*						
Age, years*	60 (53-64)	67 (52-74)	0.16	59 (48-64)	68.5 (54-75)	0.01
Male sex (%)	11 (61)	60 (63)	0.87	17 (63)	54 (63)	0.99
Fatal outcome (%)	11 (61)	32 (34)	0.03	17 (63)	26 (30)	0.002
Length of stay, days*	23 (20-45)	15 (8-41)	0.03	23 (13-45)	14.5 (7-41)	0.03
*Comorbid conditions*						
Immune suppression (%)	18 (100)	76 (80)	0.04	27 (100)	67 (78)	0.006
Neutropenia (%)	15 (83)	23 (24)	<0.001	17 (63)	21 (24)	<0.001
Cardiovascular disease (%)	7 (39)	59 (62)	0.07	11 (41)	55 (64)	0.03
Diabetes (%)	5 (28)	32 (34)	0.62	7 (26)	30 (35)	0.39
Pulmonary disease (%)	1 (6)	10 (11)	1	1 (4)	10 (12)	0.46
Neurological disease (%)	2 (11)	22 (23)	0.35	6 (22)	18 (21)	0.89
Renal disease (%)	0 (0)	11 (12)	0.21	1 (4)	10 (12)	0.46
Haematological cancer (%)	17 (94)	19 (20)	<0.001	20 (74)	16 (19)	<0.001
Charlson Comorbidity Score*	2 (2-3)	3 (1-5)	0.86	2 (2-3)	3 (2-5)	0.27
*Patients clinical record*						
SAPS II*	39.5 (36-44)	32 (23-41)	0.002	37 (28-43)	32 (24-41)	0.57
AET (%)	8 (44)	66 (70)	0.04	11 (41)	63 (73)	0.002
ADT (%)	13 (72)	83 (87)	0.14	19 (70)	77 (90)	0.03

*Median (interquartile range).

MBL-PA, metallo-β-lactamase producing *Pseudomonas aeruginosa*; 3/4MDR-PA, 3/4MDR-*Pseudomonas aeruginosa;* SAPS II, Simplified Acute Physiology Score II; AET, appropriate empirical treatment; ADT, appropriate definitive treatment.

### Clinical outcomes

Table 4 shows the results of the univariate analysis. While BSIs with 3/4MDR-PA and *P. aeruginosa* strains resistant to meropenem (MEM-resistant PA) were significantly associated with mortality (HR 1.98, *P* = 0.03, and HR 1.98, *P* = 0.03, respectively), this relationship was less conclusive for MBL-PA (HR 1.83, *P* = 0.1) and ceftazidime resistant *P. aeruginosa* (CAZ-resistant PA, HR 1.88, *P* = 0.06). Ciprofloxacin resistant *P. aeruginosa* (CIP-resistant PA) and piperacillin-tazobactam resistant *P. aeruginosa* (TZP-resistant PA) showed no significant impact on mortality (*P* = 0.43 and 0.28, respectively). Of note, appropriate empirical treatment turned out not to be a predictor of mortality (*P* = 0.52). Neither MBL-PA nor 3/4MDR-PA or *P. aeruginosa* strains with a resistance to certain antimicrobial agents (MEM, CAZ, CIP, TZP) had an effect on length of stay (LOS) when the entire cohort was investigated. Including only the survivors in the analysis (n = 70), a weak association between MBL-PA, 3/4MDR-PA and LOS became apparent (*P* = 0.08 and *P* = 0.06, respectively), indicating an extended LOS for those patients infected with the multidrug-resistant strains (HR 0.49 and 0.55, respectively).

**Table 4:** **Univariate analysis: Hazard ratios for deaths and discharge (dead or alive) in patients with** ***Pseudomonas aeruginosa*** **BSI**

**Exposure**	**Hazard ratio for deaths (95% CI)**	**P-value**	**Hazard ratio for discharge–time adjusted (95% CI)**	**P-value**	**Hazard ratio for discharge–time adjusted (95% CI) only survivors (n = 70)**	**P-value**
*Basic parameter*						
Male sex	1.02 (0.55-1.88)	0.95	1.14 (0.77-1.68)	0.5	1.28 (0.77-2.12)	0.32
Age, years	1.0073 (0.9889-1.0261)*	0.43	1.0164 (1.0039-1.0291)*	0.007	1.0155 (0.9938-1.033)*	0.07
Nosocomial infection	2.14 (1.05-4.27)	0.03	0.67 (0.42-1.07)	0.1	0.52 (0.28-0.97)	0.04
*Origin of bacteremia*						
Unknown (primary BSI)	1.07 (0.57-2)	0.83	1.85 (1.24-2.75)	0.003	2.22 (1.32-3.73)	0.003
Secondary BSI	1.25 (0.67-2.31)	0.47	0.79 (0.53-1.16)	0.24	0.76 (0.46-1.25)	0.29
VC BSI	0.22 (0.03-1.66)	0.06	0.39 (0.18-0.85)	0.008	0.37 (0.15-0.89)	0.01
*Comorbid conditions*						
Immune suppression	2.31 (0.71-7.55)	0.12	0.54 (0.31-0.94)	0.04	0.5 (0.26-0.96)	0.04
Haematological cancer	1.23 (0.67-2.28)	0.5	0.61 (0.39-0.93)	0.02	0.5 (0.27-0.91)	0.02
Chemotherapy	0.83 (0.45-1.56)	0.58	0.62 (0.41-0.94)	0.02	0.6 (0.35-1.03)	0.06
Cardiovascular disease	0.65 (0.36-1.2)	0.18	0.77 (0.52-1.14)	0.2	0.83 (0.49-1.38)	0.48
Diabetes	1.43 (0.77-2.65)	0.25	0.78 (0.51-1.19)	0.25	0.55 (0.31-0.99)	0.04
Charlson Comorbidity Score	0.96 (0.83-1.11)*	0.66	1 (0.92-1.1)*	0.83	1.01 (0.9-1.13)*	0.77
*Patients clinical record*						
SAPS II	1.0375 (1.0184-1.057)*	<0.001	0.9961 (0.9828-1.0097)*	0.58	0.974 (0.9557-0.9926)*	0.005
Neutropenia	1.41 (0.77-2.58)	0.27	0.62 (0.4-0.94)^†^	0.02	0.43 (0.23-0.79)	0.004
Chemotherapy	0.83 (0.45-1.56)	0.58	0.62 (0.41-0.94)	0.02	0.6 (0.35-1.03)	0.06
Steroids	1.82 (0.86-3.84)	0.1	0.46 (0.3-0.7)	<0.001	0.3 (0.17-0.54)	<0.001
Concomitant infections	1.22 (0.56-2.71)	0.59	0.27 (0.16-0.44)	<0.001	0.23 (0.11-0.46)	<0.001
Recent surgery	0.65 (0.34-1.22)	0.18	0.54 (0.35-0.81)	0.003	0.47 (0.27-0.82)	0.007
AET	0.81 (0.44-1.5)	0.52	0.89 (0.59-1.34)	0.6	0.9 (0.53-1.51)	0.7
ADT	0.33 (0.16-0.68)	0.007	0.44 (0.25-0.77)	0.008	0.4 (0.17-0.92)	0.052
*Pathogen exposure*						
MBL-PA	1.83 (0.92-3.64)	0.1	0.75 (0.44-1.27)	0.27	0.49 (0.21-1.17)	0.08
3/4MDR-PA	1.98 (1.07-3.67)	0.03	0.76 (0.49-1.19)	0.23	0.55 (0.25-1.07)	0.06
MEM-resistant PA	1.98 (1.08-3.62)	0.03	0.85 (0.55-1.3)	0.45	0.64 (0.33-1.23)	0.17
CAZ-resistant PA	1.88 (0.99-3.57)	0.06	0.72 (0.45-1.16)	0.17	0.53 (0.25-1.15)	0.09
CIP-resistant PA	1.27 (0.69-2.34)	0.43	0.93 (0.62-1.39)	0.73	0.78 (0.45-1.34)	0.38
TZP-resistant PA	1.41 (0.76-2.61)	0.28	0.78 (0.52-1.19)	0.26	0.68 (0.37-1.23)	0.2

^†^Proportional hazard assumption not fulfilled for this parameter.

*Per 1 unit increase.

95% CI, 95% Confidence interval; BSI, blood stream infection; VC BSI, vascular catheter-related blood stream infection; SAPS II, Simplified Acute Physiology Score; AET, appropriate empirical treatment; ADT, appropriate definitive treatment; MBL-PA, metallo-β-lactamase producing *Pseudomonas aeruginosa*; 3/4MDR-PA, 3/4MDR-*Pseudomonas aeruginosa*; PA, *Pseudomonas aeruginosa*; MEM, Meropenem; CAZ, Ceftazidime; CIP, Ciprofloxacin; TZP, Piperacillin-tazobactam.

In multivariate models, the SAPS II score (HR 1.046), cardiovascular disease (HR range 0.44-0.46) and appropriate definitive treatment (HR range 0.25-0.26) were significantly associated with mortality. After adjusting for these confounders, neither MBL-PA nor 3/4MDR-PA remained associated with mortality (Table 5). Also, resistance to either meropenem, ceftazidime, ciprofloxacin or piperacillin-tazobactam on its own was not associated with mortality (data not shown). Furthermore, we did not observe a significant influence of MBL-PA or 3/4MDR-PA on LOS in the fully adjusted models (Table 5). Instead, the presence of concomitant infections was a significant predictor of discharge in all models (HR < 1).

**Table 5:** Multivariate analysis: Hazard ratios for deaths and discharge (dead or alive)

**Parameter**	**Hazard ratio for deaths (95% CI)**	**P-value**	**Hazard ratio for discharge–fully adjusted (95% CI)**	**P-value**	**Hazard ratio for discharge–fully adjusted (95% CI) only survivors (n = 70)**	**P-value**
MBL-PA	0.98 (0.45-2.1)^a^	0.97	1.28 (0.63-2.58)^c^	0.49	1.53 (0.53-4.45)^e^	0.44
3/4MDR-PA	1.37 (0.68-2.72)^b^	0.37	1.24 (0.69-2.21)^d^	0.46	0.75 (0.31-1.83)^f^	0.53

^a^Significant in the same model: SAPS II (HR 1.046, *P* < 0.001), cardiovascular disease (HR 0.44, *P* = 0.015) and ADT (HR 0.25, *P* = 0.002).

^b^Significant in the same model: SAPS II (HR 1.046, *P* < 0.001), cardiovascular disease (HR 0.46, *P* = 0.025) and ADT (HR 0.26, *P* = 0.003).

^c^Significant in the same model: Concomitant infection (HR 0.3, *P* < 0.001), ADT (HR 0.39, *P* = 0.004).

^d^Significant in the same model: Concomitant infection (HR 0.27, *P* < 0.001), ADT (HR 0.4, *P* = 0.007).

^e^Significant in the same model: Concomitant infection (HR 0.32, *P* = 0.002), vascular catheter-related BSI (HR 0.41, *P* = 0.04), nosocomial infection (HR 0.44, *P* = 0.04).

^f^Significant in the same model: Concomitant infection (HR 0.32, *P* = 0.002), neutropenia (HR 0.39, *P* = 0.006), nosocomial infection (HR 0.37, *P* = 0.004), ADT (HR 0.25, *P* = 0.008).

95% CI, 95% Confidence interval; MBL-PA, metallo-β-lactamase producing *Pseudomonas aeruginosa*; 3/4MDR-PA, 3/4MDR-*Pseudomonas aeruginosa*; SAPS II, Simplified Acute Physiology Score; ADT, appropriate definitive treatment.

### Multi locus sequence typing results

To investigate whether the results could be influenced by the genetic relatedness of our strains, we performed molecular typing of all 27 isolates exhibiting a multidrug-resistant phenotype (3/4MDR). Ten different sequence types were observed (data not shown). Of note, the fourteen IMP-8 producing isolates belonged to the sequence type 308. VIM-2 producing isolates belonged to the sequence types 233 (n = 3) and 395 (n = 1). Non-3/4MDR-PA (n = 86) showed a wide variety of resistance phenotypes and were therefore considered to have a low degree of genetic relatedness.

## Discussion

Despite the extensive spread of MBL-producing organisms, the influence of such pathogens on clinical outcomes has not been comprehensively assessed. The present study showed that MBL-PA BSI results in a higher in-hospital mortality than BSI with Non-MBL-PA. These results are in line with earlier reports [14-16]. However, BSI with MBL-PA did not turn out to be an independent predictor of mortality. The higher mortality in the MBL-PA group seemed mainly mediated by the severity of the underlying diseases, as previously reported by Zavascki and colleagues in patients with various nosocomial infections [16]. While the administration of appropriate definitive treatment had independently a strong protective effect (HR range 0.25-0.26), there was only a weak tendency that patients with MBL-PA BSI were less likely to have received sufficient therapy. After the emergence of the first MBL-PA isolates, colistin (colistimethate sodium) was added to the empirical therapeutic regime when a BSI with *P. aeruginosa* was suspected. MBL-PA isolates were resistant to all antimicrobial agents except for colistin. In fact, colistin was intravenously administered to 13 of 18 patients with MBL-PA BSI (72%), and these patients were considered to have received appropriate therapy. It can be speculated that colistin has improved the outcome of these patients in our setting. This hypothesis is supported by the fact that the treatment variable for definitive therapy proved to be strongly protective when regarding colistin as appropriate treatment in the multivariate models. However, our study was not designed to resolve this issue. And despite the growing evidence for the therapeutic benefit of intravenous colistin in adult patients infected with multidrug-resistant *P. aeruginosa*[30-32], prospective and randomized control trials are required to prove its efficacy and safety.

Different phenotypes of resistance of *P. aeruginosa* in BSI did not prove to be independent predictors of mortality, either. In the case of 3/4MDR-PA, the observed difference in mortality compared to Non-3/4MDR-PA (63% vs 30%, *P* = 0.002, Table 3) appeared to be due to the lower chance of patients with 3/4MDR-PA BSI to have received appropriate definitive treatment. This emphasizes the importance of reevaluating the initial therapeutic regimes in a hospital setting with a relevant incidence of multidrug-resistant *P. aeruginosa*. It also stresses the importance of susceptibility testing results from the associated microbiology laboratories and the need to effectively and appropriately adjust empirical treatments where necessary. Whether multidrug-resistant phenotypes of *P. aeruginosa* have an intrinsic influence on the risk of a lethal outcome is still controversial. While some studies revealed infection with multidrug-resistant *P. aeruginosa* to be an independent predictor of mortality [3,33,34] others have not observed such a connection [35,36]. The reasons for this heterogeneity are unknown. However, one possibility is that not all of these studies performed molecular genotyping, and their results could have been influenced by the presence of just a few dominant strains, possibly in possession of virulence factors that could have contributed to worse clinical outcomes [37]. These or similar circumstances may have led to confounding effects. Although we did not perform molecular typing for all isolates, the identification of 10 different MLST sequence types among the multidrug-resistant phenotypes (3/4MDR-PA) in our study makes such a confounding less likely, with the exception of the 18 MBL-PA isolates which belong to three different strains. These strains circulated in only a few wards at the tertiary teaching hospital, suggesting that transmission within the hospital is still a major factor for the spread of MBL-PA. Another reason for our observations might be the limitation in study power. Since we needed to get by with less than 10 outcome events per variable in our multivariate models, we may have been incapable of detecting a minor effect of MBL producers or resistant phenotypes on mortality. Nevertheless, a relevant influence on mortality is unlikely to have been overlooked. Larger-scaled, multicenter studies are still required to investigate this issue and to generalize results to other settings with confidence.

The absence of a significant relation between an appropriate empirical treatment (within 24 hours) and mortality is another interesting finding. Lodise and colleagues noted that the risk of a fatal outcome rises in patients with *P. aeruginosa* BSI once the delay in receiving appropriate treatment exceeded 52 hours [36]. However, their study did not provide information about the probable origin of infection. This might be an important point since interventions like abolition of urinary obstructions and removal of catheters are the mainstay of therapy in blood stream infections with the urinary tract or a vascular catheter as probable origins of infection, leaving prompt antimicrobial treatment no more than a secondary role in the clinical management. This subpopulation of our patients (origin of infection: 16% urinary tract and 7% vascular catheter-related, Table 1) may have influenced the results in the observed direction. It must be noted that this proportion may be an underestimation due to our study’s limitation as a retrospective investigation. However, we excluded patients among whom vital clinical information was missing (4 of 120 patients) and are thus confident that the data accurately represents the real situation to a high degree.

In addition, we investigated whether MBL production or resistance phenotypes were associated with the length of stay. BSI with a MBL producer or resistant phenotype did not prolong the LOS, while the presence of concomitant infections besides a PA-BSI did so (HR < 1, all models). In fully adjusted models wherein only survivors were included, nosocomial infections were also significantly associated with a prolonged stay. These results indicate that any concomitant infection should be avoided to reduce additional morbidity and hospital costs, leading to the demand for strict routine infection control measures.

## Conclusions

The administration of appropriate definitive treatment in patients with PA-BSI, even when delayed, remains the backbone for improving clinical outcomes and should be combined with routine infection control measures to further reduce morbidity and costs. The pattern of resistance within a hospital should be continuously monitored for a rapid detection of changes and consecutive adjustment of empirical treatment regimes.

## Competing interests

Our study was supported in part by the German Centre of Infection Research (DZIF). The funder had no role in study design, data collection and analysis, decision to publish, or preparation of the manuscript. The authors declare that they have no competing interests.

## Authors’ contributions

MW led the investigation, including design of the cohort study. IK, MM, KS, FH, WV, IF and UM collected clinical and epidemiological data. Laboratory analysis was conducted by SP, MM and IBA. MW and SP analyzed the data and wrote the manuscript. All authors read, commented on and approved the final manuscript.

## Pre-publication history

The pre-publication history for this paper can be accessed here:

http://www.biomedcentral.com/1471-2334/13/515/prepub
